# Foraging behavior links climate variability and reproduction in North Pacific albatrosses

**DOI:** 10.1186/s40462-015-0050-9

**Published:** 2015-10-01

**Authors:** Lesley H. Thorne, Elliott L. Hazen, Steven J. Bograd, David G. Foley, Melinda G. Conners, Michelle A. Kappes, Hyemi M. Kim, Daniel P. Costa, Yann Tremblay, Scott A. Shaffer

**Affiliations:** School of Marine and Atmospheric Sciences, Stony Brook University, Stony Brook, NY 11790 USA; Environmental Research Division, Southwest Fisheries Science Center, NOAA Fisheries, 99 Pacific St., Suite 255A, Monterey, CA 93940 USA; Cooperative Institute for Marine Ecosystems and Climate, University of California Santa Cruz, 100 Shaffer Road, Santa Cruz, CA 95060 USA; Department of Ocean Sciences, University of California Santa Cruz, 100 Shaffer Road, Santa Cruz, CA 95060 USA; Department of Fisheries and Wildlife, Oregon State University, 104 Nash Hall, Corvallis, OR 97331-3803 USA; Department of Ecology and Evolutionary Biology, University of California Santa Cruz, 100 Shaffer Road, Santa Cruz, CA 95060 USA; Institut de Recherche pour le Développement (IRD), Research Unit Marine Biodiversity, Exploitation and Conservation UMR248 MARBEC, Avenue Jean Monnet, CS 30171 - 34203 Sète Cedex, France; Department of Biological Sciences, San José State University, One Washington Square, San Jose, CA 95192 USA; Institute of Marine Sciences, University of California Santa Cruz, 100 Shaffer Road, Santa Cruz, CA 95060 USA

**Keywords:** Albatross, Movement, Reproductive success, Climate, Environmental variability

## Abstract

**Background:**

Climate-driven environmental change in the North Pacific has been well documented, with marked effects on the habitat and foraging behavior of marine predators. However, the mechanistic linkages connecting climate-driven changes in behavior to predator populations are not well understood. We evaluated the effects of climate-driven environmental variability on the reproductive success and foraging behavior of Laysan and Black-footed albatrosses breeding in the Northwest Hawaiian Islands during both brooding and incubating periods. We assessed foraging trip metrics and reproductive success using data collected from 2002–2012 and 1981–2012, respectively, relative to variability in the location of the Transition Zone Chlorophyll Front (TZCF, an important foraging region for albatrosses), sea surface temperature (SST), Multivariate ENSO Index (MEI), and the North Pacific Gyre Oscillation index (NPGO).

**Results:**

Foraging behavior for both species was influenced by climatic and oceanographic factors. While brooding chicks, both species traveled farther during La Niña conditions, when NPGO was high and when the TZCF was farther north (farther from the breeding site). Models showed that reproductive success for both species showed similar trends, correlating negatively with conditions observed during La Niña events (low MEI, high SST, high NPGO, increased distance to TZCF), but models for Laysan albatrosses explained a higher proportion of the variation. Spatial correlations of Laysan albatross reproductive success and SST anomalies highlighted strong negative correlations (>95 %) between habitat use and SST. Higher trip distance and/or duration during brooding were associated with decreased reproductive success.

**Conclusions:**

Our findings suggest that during adverse conditions (La Niña conditions, high NPGO, northward displacement of the TZCF), both Laysan and Black-footed albatrosses took longer foraging trips and/or traveled farther during brooding, likely resulting in a lower reproductive success due to increased energetic costs. Our results link climate variability with both albatross behavior and reproductive success, information that is critical for predicting how albatross populations will respond to future climate change.

## Background

Top predators in marine systems have shown marked declines in recent years due to the combined effects of overfishing, bycatch, and climate change [1–4]. Upper trophic level predators play a key role in marine ecosystems, and declines in these species can lead to a wide range of direct and indirect consequences, including trophic cascades [5–9]. Predictions suggest that there will be both winners and losers under climate change projections in the North Pacific [10], but few studies have examined how behavioral plasticity mediates top predator responses to climate change.

The effects of climate change on marine systems are predicted to surpass ecological tipping points in the future [11, 12], with significant effects forecasted for habitats used by a number of marine predators [10, 13]. Many studies linking climate to demographic change in marine predators have focused on responses to broad-scale variability such as changes in ice cover [e.g., 14–17]. However, more subtle changes such as habitat shifts can have important population-level effects and have received less attention in the literature. Changes in the location of foraging habitats can make them inaccessible or energetically costly to reach, which can have important implications for the foraging efficiency and population trajectories of marine predators [18, 19]. A quantitative understanding of the biological mechanisms linking climate to population change is integral to predicting responses of marine predators to future climate-driven ecosystem change [14].

Albatrosses are ideal species to examine the behavioral and demographic implications of climate variability for marine predators. Highly mobile seabirds such as albatrosses are easily monitored, integrate food resources across a large area, and can be used to indicate changes in the abundance, size and availability of their prey species, as well as underlying oceanographic drivers of these changes [e.g., 20–23]. Albatrosses and other pelagic seabirds have the ability to search for prey over vast expanses of ocean due to their low cost of gliding flight [24–27], but during breeding their at-sea movements are spatially and temporally constrained because they must return to their nest site to exchange parental duties and/or feed offspring [28–30]. Moreover, the energetic costs of reproduction vary considerably within the breeding season [31]. For example, during the incubation period, parents can take long foraging trips (~7-20 d) because their partner can fast for prolonged periods while incubating the egg. In contrast, parents brooding small chicks must regularly provision their rapidly-growing chicks, which dramatically shortens foraging trips (typically ~1-3 d) [28, 30, 32, 33]. Because trip duration is constrained by the food requirements of the chick, albatrosses brooding chicks are unable to exploit the more distant foraging areas used during the incubation period. Consequently, oceanographic variability has the potential to limit the accessibility of foraging habitat for breeding albatrosses, especially during the brooding period. Thus, identifying how this variability influences albatross behavior when parents are most constrained (i.e., during brooding) could shed light on the link between changes in ocean climate and demographic effects on albatross populations.

**Fig. 1:**
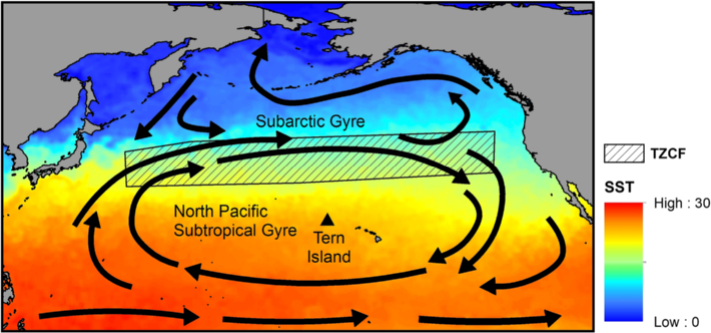
Location of Tern Island relative to North Pacific subtropical and subarctic gyres and the Transition Zone Chlorophyll Front (TZCF)

Central place foragers, such as albatrosses during breeding, have limited dispersal and show distant-dependent costs of accessing resources [34]. Consequently, we would expect that foraging areas that are predictable in time and space would be particularly important to these animals. Within the North Pacific, variability in the location of seasonally predictable oceanographic features such as the Transition Zone Chlorophyll Front (TZCF) affects both the distribution and survival of marine predators [35, 36]. The TZCF migrates seasonally over more than 1000 km north-south (Fig. 1), typically reaching its southernmost latitude in February and its northernmost latitude in August [37]. Interannual variability in the location of the TZCF is driven by broad-scale climatic variability; for example, a stronger Aleutian low elicits a southward shift of westerlies, stronger mixing in the transition zone, and contributes to a southern displacement of the TZCF [37]. In contrast, the TZCF is displaced northward with greater meandering during La Niña events. Displacement of the TZCF can influence its utility as foraging habitat for top predators; for example, Hawaiian monk seals showed increases in pup mass and girth during El Niño events, likely because of closer proximity to the subtropical front and cooler, more productive waters during El Niño years [38]. Climate models predict an expansion of the subtropical gyre and northward shift in the TZCF over the next century with potential impacts to top predators including Pacific albatrosses [10, 39].

**Fig. 2:**
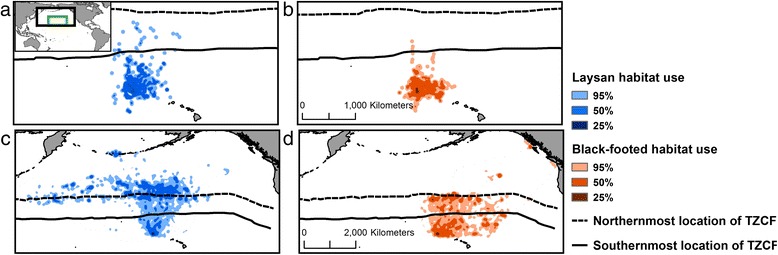
Habitat used by albatrosses relative to the location of the TZCF. Kernel density distributions (25, 50 and 95 %) are shown for brooding (**a**, **b**; extent indicated in green) and incubating (**c**, **d**; extent indicated in black) Laysan and Black-footed albatrosses, respectively. The TZCF is shown at its northermost (Nov. 4) and southermost (Feb. 25) location during the 2008/2009 incubating and brooding periods to demonstrate variability in TZCF location within these breeding stages. Note that the TZCF is located further south outside of the albatross breeding season

In the Northwest Hawaiian Islands (hereafter NWHI), breeding Laysan (*Phoebastria immutabilis*) and Black-footed (*P. nigripes*) albatrosses show considerable interannual variability in reproductive success. Laysan and Black-footed albatrosses also exhibit spatial differences in their foraging distribution; Laysans forage farther north and west in the subarctic gyre, whereas Black-footeds forage primarily farther south and east in the subtropical gyre [29, 33, 40; Fig. 2]. We hypothesize that the proximity of the TZCF to albatross breeding sites, as influenced by large-scale climate modes, affects the behavior and reproductive success of these species. Specifically, we predict that northward displacement of the TZCF and increases in SST in albatross habitat during La Niña events are associated with increased trip distance and duration and decreased reproductive success. We posit that these effects have the greatest impact when parents are brooding small chicks. Since Laysan albatrosses use waters north of the TZCF more frequently than Black-footed albatrosses [29, 33, 40, Fig. 2], we suggest that displacement of the TZCF will have more pronounced effects on Laysan albatrosses.

This study has three important differences from previous studies investigating that habitat use of North Pacific albatrosses [29, 33]. First, we use telemetry data collected over a ten year period, allowing connections with large-scale climatic variability (e.g., MEI, NPGO) to be evaluated in detail. Secondly, we evaluate habitat use during both the brooding and incubating periods, whereas previous multi-year studies of North Pacific albatrosses examined only incubating tracks [33]. Lastly, we advance previous studies by linking analyses of telemetry data and oceanographic and climatic data with reproductive success to examine how environmental variability and trip metrics influence reproduction in North Pacific albatrosses.

## Methods

### Study species

Laysan and Black-footed albatrosses breed sympatrically on atolls throughout the NWHI and show periodic declines in their reproductive success. The breeding cycle of both Laysan and Black-footed albatrosses is well defined; eggs are laid in November and December, and the incubation period lasts for approximately two months. Adult albatrosses then brood chicks for several weeks (late January through mid to late February), guard and provision the chicks for approximately one month and then return to the breeding site to feed them regularly before the chicks fledge in June or July [41, 42]. Approximately 4300 Black-footed albatrosses and 3200 Laysan albatrosses nest at Tern Island, representing 6.9 and 0.5 % of the total population in the North Pacific, respectively [43]. The albatross colony at Midway Atoll is much larger, comprising approximately 408,000 Laysan and 22,000 Black-footed albatrosses [43]. However, standardized surveys of the colony at Midway Atoll were initiated much later than those at Tern Island (1990s on Midway vs. 1980 on Tern Island) and the resulting time series is considerably shorter.

### Albatross telemetry and reproductive success data

We examined the foraging behavior and reproductive success of Laysan and Black-footed albatrosses on Tern Island, French Frigate Shoals, NWHI (23.87°N, 166.28°W). Data on albatross reproductive success at Tern Island (defined as the number of chicks fledged per eggs laid) were obtained from the United States Fish and Wildlife Service (USFWS). Standardized surveys of breeding birds and active nests have been conducted at Tern Island since 1980; we used data from 1981-2012 (November-February 1981/82-2011/12) to be consistent with available SST data. Reproductive success data are referred to by the January/February calendar year (e.g., data from the November–February 1981/1982 breeding season are referred to as data from 1982). Albatross nests on Tern Island were assigned to survey plots and were numbered and monitored by USFWS personnel throughout each breeding season. Chicks were banded, and each nest was monitored weekly for hatching chicks, chicks present within 30 m of the nest site, and dead chicks; chicks were assumed to be dead if not found within 30 m of the nest site for three successive observations.

Nest desertion by adults is the primary cause of reproductive failure in Laysan and Black-footed albatrosses, and can be associated with poor foraging conditions, death of the adults, or inexperienced breeders. Short-term climatic events such as flooding and storms can also influence reproductive success over short time periods [44, 45, USFWS unpublished data]. However, given the protracted breeding season of Laysan and Black-footed albatrosses (from November to June), such short-term events are unlikely to influence reproductive success of the entire colony. At Tern Island, reproductive success of these species appears to show periodic declines during which reproductive success remains depressed for 2–3 years. We suggest that this pattern of reproductive success is consistent with long-term oceanographic variability, and we assess this hypothesis below.

We recorded albatross movements during the incubation and brooding periods from 2002–2006 and from 2008–2012 using satellite platform terminal transmitters (30 g Pico-100, Microwave Telemetry, Columbia, MD, 42 g SPOT4 and SPOT5, Wildlife Computers, Redmond, WA) and GPS data loggers (40 g Technosmart GPS, 35 g TechnoSmart GiPSy, 32 g E&O Technologies, and 30 g igotU, GT-120, Mobile Action Technology Inc, Taiwan). Tags were attached with Tesa adhesive tape (Tesa, Hamburg, Germany) to dorsal feathers. All tag weights represent water-proofed packaged tags and are well below the maximum mass threshold recommended for albatrosses [46]. Only complete trips in which tracks covered the entire trip (leaving from and retuning to Tern Island) were included in the analysis; a total of 93 trips for Laysan and 97 trips for Black-footed albatrosses were included in the analysis (Table 1), and only one trip per individual was tracked and included in the analysis. Trip duration decreased towards the end of the incubation period for both species; we therefore included only incubating trips during the first two months of the incubation period (November and December), when trip duration remained consistent (Pearson’s correlation coefficient between trip duration and day of breeding season <0.15, *p* value > 0.4 for both species). Trip duration was consistent throughout the brooding period (late January through late February) for both species (Pearson’s correlation coefficient between trip duration and day of breeding season < 0.15, *p* value > 0.3 for both species). GPS and PTT tag data were resampled to a six-hour time scale in order to provide sufficiently detailed spatial information at a time scale that was appropriate for both tag types. Resampling was conducted using the Minimum Covariance Determinant (MCD) in the MASS library (version 7.3-31) of the R statistical package (version 3.0.2) in order to provide a robust estimate of location at each time step that is not strongly influenced by outliers occurring due to the spatial resolution of telemetry data. When fewer than four locations were available within a time window, MCD cannot be computed and the coordinate-wise median was used [47].

**Table 1:** Tracks used in analyses by species, breeding stage, tag type and year

Species	Laysan				Black-footed			
Stage	Incubating		Brooding		Incubating		Brooding	
Year	GPS	PTT	GPS	PTT	GPS	PTT	GPS	PTT
2003	0	1	0	3	0	5	0	4
2004	0	7	0	0	0	6	0	0
2005	1	8	0	14	0	4	0	12
2006	0	4	0	12	0	2	0	11
2007	0	0	0	0	0	0	0	0
2008	0	2	1	9	0	2	1	6
2009	0	0	9	0	1	3	11	0
2010	0	5	4	1	0	4	6	0
2011	2	5	0	0	5	3	0	0
2012	1	2	9	0	3	4	5	0
TOTAL	4	34	23	39	9	33	23	33

For each albatross foraging trip, we assessed cumulative trip distance, maximum distance travelled from Tern Island, and the duration of each trip using the ArgosFilter (version 0.63) and MASS libraries in R. To examine albatross movement in relation to the location of the TZCF, we calculated distances to TZCF for each location on each track using daily rasters of distance to TZCF (see below) and assessed which tagged birds spent time north of the TZCF. We produced kernel density distributions for both species during incubating and brooding periods. Kernel densities were calculated with ArcGIS 10.2.2 Spatial Analyst using a fixed radius of 100 km for incubating trips and 50 km for brooding trips. We then delineated habitat use as the 95, 50 and 25 % isopleths of kernel density distributions [29, 33].

### Transition Zone Chlorophyll Front (TZCF)

The TZCF is a basin-wide front, spanning more than 8000 km from west to east across the North Pacific. Representing a zone of convergence, the TZCF separates the cool, well-mixed, nutrient-rich waters of the subarctic gyre from the warmer, stratified, nutrient-poor waters of the subtropical gyre [35]. The TZCF is characterized by a sharp chlorophyll gradient, and is defined by a surface chlorophyll value of 0.2 mg/m^3^ [35]. The 18 °C isotherm in SST has been demonstrated to provide a proxy for the location of the TZCF [37].

During the breeding season of albatrosses in the North Pacific (November–June), there is considerable interannual variability in the location of the TZCF [37] but the front is generally closest to Tern Island in January and February, coinciding with the brooding period (Fig. 2). Given the importance of the TZCF as feeding grounds for breeding albatrosses, the proximity of the front to the NWHI likely has important implications for both Laysan and Black-footed albatrosses. Historical records of SST are available at a finer temporal and spatial resolution compared to chlorophyll and can thus be more useful in historical models of habitat use [37]. We localized the TZCF on a daily time scale using daily Group for High Resolution SST (GRHSST) images with a 5 km resolution. We created shapefiles of TZCF location for each day of the incubating and brooding periods from 1982–2012 (November-February 1981/1982-2011/2012) and generated rasters of distance to TZCF for each day in order to examine variability in frontal location relative to albatross reproductive success and foraging distribution. Within the albatross brooding period, the TZCF can range from a minimum of approximately 400 km to a maximum of approximately 1150 km from Tern Island. All spatial analyses were conducted in ArcGIS 10.2.2 using the Spatial Analyst extension and using the raster (version 2.3-34), geosphere (version 1.3-8) and Imap (version 1.32) R Statistical packages [48].

### Large-scale Oceanographic and Climatic Indices

We obtained time series of the North Pacific Gyre Oscillation index (NPGO) and the Multivariate El Niño Southern Oscillation (ENSO) Index (MEI) to represent large-scale climatic and oceanographic variability in the central North Pacific (MEI and NPGO data obtained from http://www.esrl.noaa.gov/psd/enso/mei/table.html and http://www.o3d.org/npgo/npgo.php, respectively). NPGO represents the strength of the North Pacific Gyre [49, 50], where high/low values indicate expansion/contraction of the gyre, respectively. MEI identifies El Niño events by incorporating variability in six oceanographic and climatic variables over the tropical Pacific [51], with positive values of MEI representing El Niño conditions, and negative values representing La Niña conditions. El Niño events have been linked with southern migrations in the TZCF [37] and the front migrates in association with expansion/contraction of the North Pacific gyre, making MEI and NPGO important variables to evaluate in our models of albatross behavior and reproductive success.

We used GRHSST data (see above) to examine SST on an annual scale relative to reproductive success, and at a daily scale relative to albatross trip metrics. The location of the TZCF represents the boundary between the subarctic and subtropical gyres and therefore reflects broad-scale patterns in SST, but variability in SST at a finer spatial scale may influence search effort of foraging albatrosses [33] and was therefore examined separately. We produced rasters of mean SST for each year during the albatross incubating and brooding periods from 1981–2012 (November–February 1981/82–2011/12), as well as the 31-year mean for this time period. We then produced rasters of SST anomalies (SSTa) using the formula SSTa_*i*_ = SST_*i*_ - SST_mean_, where *i* is the year, SST_*i*_ is the mean SST from November–February for year *i* and SST_mean_ is 31-year SST mean from the November–February time period. As with reproductive success data, SST data are referred to by the calendar year of the January/February period (i.e., SST data from November–February 1981/1982 are referred to as 1982 SST data). We assessed SST within the 95 % kernel density isopleths for Laysan and Black-footed albatrosses during the brooding and incubating stages, respectively. There are considerable differences in habitat use between species and breeding stages, and therefore it was important to assess changes in SST in each region independently [52].

### Statistical Analyses

In order to examine the effects of oceanographic and climatic indices on albatross reproductive success, we examined average, minimum and maximum values of SST, MEI and NPGO annually during the albatross breeding season (November–February). We examined time-lagged effects on albatross reproductive success using cross-correlation functions (CCFs). CCFs are useful for identifying predictor variables (x_t_) that might have lagged effects on a dependent variable (y_t_), and examine correlations between the dependent variable and predictor variables at different time lags (correlations between x_t-h_ and y_t_ for different time lags represented by h = 0, 1, 2, 3, etc.). Here we identified variables that had time-lagged effects on albatross reproductive success for time lags of 0 to 5 years. Variables with significant CCFs were time-lagged and included as lagged variables for further analyses. However, after applying model selection (described below), time lagged variables were not included in final models. The final models of albatross reproductive success included the following variables: minimum distance to TZCF, minimum NPGO, minimum MEI and mean SST in brooding habitat. Albatross trips were examined over the ten-year period, and were evaluated relative to both broad-scale (monthly MEI and NPGO) and finer-scale (daily SST and TZCF) oceanographic and climatic variables. Though MEI and NPGO are broad-scale metrics, these variables vary considerably over a period of several months and were therefore sampled at the midpoint of albatross foraging trips along with finer-scale variables. To quantify the effects of these variables on albatross trip metrics, we assessed minimum, maximum and mean values of distance to TZCF, NPGO, MEI and SST in brooding or incubating habitat. After model selection, minimum SST was used in trip metrics for Laysan albatrosses, while mean SST was used in models for Black-footed albatrosses.

We examined relationships between La Niña events, gyre expansion, TZCF location and SST on albatross trip metrics and reproductive success using several analytical approaches. We used Pearson’s correlation coefficients and Wilcoxon signed rank tests to examine relationships among different oceanographic variables and between trip metrics and reproductive success. We applied Principal Component Analysis (PCA) combined with Generalized Linear Models (GLMs) to examine the overall effects of multiple climatic indices. PCA provides a means of summarizing the variability across a number of correlated variables (MEI, NPGO, SST, proximity of TZCF to Tern Island) into fewer independent, orthogonal axes. GLMs are statistical regression models [53] that allow for different types of predictors (continuous, binary, ordinal) to be evaluated [e.g., 54]. Here we constructed separate PCAs to summarize environmental variation and to evaluate their effects on Laysan and Black-footed albatrosses, respectively, at two scales: at an annual level to evaluate effects on reproductive success, and at the trip level to assess effects on albatross trip metrics. We then used GLMs to evaluate how reproductive success and trip metrics were influenced by the environment (PC axes), and included PC axes with greater than 15 % of variance explained as predictor variables. We examined the two species’ brooding and incubating periods separately due to the differences in the timing, spatial habitat use of albatrosses, and associated differences in environmental variables. This allowed us to resolve how relationships with predictor variables differed between the brooding and incubating periods, thus indicating how metrics of albatross foraging differed during the most constraining period of breeding (brooding). For all GLMs, we used Akaike Information Criterion (AIC) [55] to select variables for the most parsimonious model [56]. PCAs and GLMs were performed using the stats (version 3.0.2) and mgcv (version 1.7-29) R Statistical packages, respectively.

To further examine relationships between albatross trip metrics and proximate oceanographic variables, we compared trip metrics with MEI, NPGO, SST, and distance to TZCF (assessed as values above/below the mean values) using Wilcoxon signed rank tests. We also compared trip metrics below/above mean reproductive success using Wilcoxon signed rank tests.

We used spatial correlations to illustrate how the relationship between SSTa and albatross reproductive success varied spatially. Using the 31-year time series for both reproductive success and SSTa (November–February average for each year from 1981–2012) described above, annual reproductive success was correlated with each SSTa grid cell using Pearson’s correlation coefficients, producing a spatial correlation between these two datasets for each grid cell.

## Results

### Variability in oceanographic and climatic variables

**Fig. 3:**
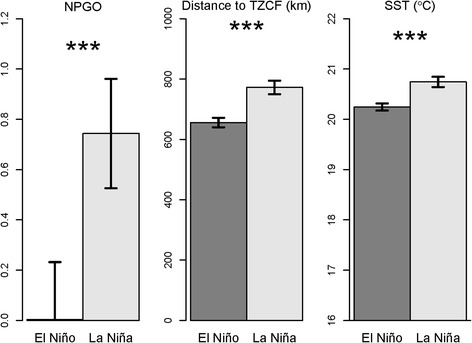
NPGO, distance to TZCF from Tern Island and SST during El Niño and La Niña conditions. Mean monthly values were evaluated in Laysan albatross brooding habitat for MEI values greater than (El Niño) or less than(La Niña) 0 , respectively, during the albatross incubating and brooding periods (November–February) from 1982–2012

The study area showed considerable variability in the oceanographic and climatic metrics over the 31-year period, both within and among years. Minimum and maximum values of these metrics are shown in Table 2, along with correlations among predictor variables. PCA loadings for environmental data are shown in Table 3. Data were calculated at the annual level and mean SST was calculated from Laysan albatross and Black-footed albatross brooding habitats, respectively. During La Niña conditions, NPGO was higher, the TZCF was located further north, and SST was higher (Fig. 3). Note that El Niño events have different effects in the eastern North Pacific, where higher SST is associated with El Niño conditions [e.g., 57–59].

**Table 2:** Correlations between environmental variables and minimum/maximum values of variables used in PCA/GLM analyses

Correlations	MEI	NPGO	Distance to TZCF	Mean SST (Laysan)	Mean SST (Black-footed)
MEI	1.00				
NPGO	−0.60	1.00			
Distance to TZCF	−0.36	0.69	1.00		
Mean SST	−0.48	0.61	0.76	1.00	
(Laysan)					
Mean SST	−0.51	0.58	0.73	0.91	1.00
(Black-footed)					
Minimum	−1.68	−3.00	411 km	14.32 °C	20.78 °C
Maximum	3.00	2.96	1154 km	26.18 °C	24.52 °C

Correlations were evaluated at an annual time scale over a 31-year period (November–February mean values, 1982–2012); mean SST was calculated in brooding habitat of Laysan and Black-footed albatrosses, respectively

**Table 3:** Loadings and variance explained for PC axes used in analyses of reproductive success and trip metrics

Variable	MEI	NPGO	Distance to TZCF	SST^a^	Cumulative Prop. Var.
Reproductive success analyses (1981–2012)					
Laysan albatross					
PC1_LAAL, annual_	0.43	−0.52	−0.52	−0.52	0.71
PC2 _LAAL, annual_	0.79	−0.16	0.50	0.32	0.88
Black-footed albatross					
PC1 _BFAL, annual_	0.44	−0.53	−0.51	−0.52	0.69
PC2 _BFAL, annual_	0.79	−0.11	0.53	0.27	0.85
Trip level analyses (2002–2012)					
Laysan albatross- Incubating trips					
PC1 _LAAL, incubating_	0.61	0.26	−0.52	−0.54	0.53
PC2 _LAAL, incubating_	−0.28	0.75	−0.41	0.43	0.84
Black-footed albatross- Incubating trips					
PC1 _BFAL, incubating_	0.49	0.46	−0.49	−0.56	0.44
PC2 _BFAL, incubating_	−0.66	0.75	0.31	−0.19	0.76
Laysan albatross- Brooding trips					
PC1 _LAAL, brooding_	−0.57	0.43	0.41	0.57	0.49
PC2 _LAAL, brooding_	0.15	−0.26	0.91	−0.27	0.71
Black-footed albatross- Brooding trips					
PC1 _BFAL, brooding_	−0.53	0.40	0.60	−0.44	0.38
PC2 _BFAL, brooding_	−0.49	0.32	0.62	−0.32	0.64

^a^After model selection mean SST was used for models of reproductive success and Black-footed albatross trip metrics, while minimum SST was used for models of Laysan albatross trip metrics

### Oceanographic and climate effects on trip metrics

Final models for albatross trip metrics demonstrated that environmental variables and indices influenced foraging behavior (Table 4). We focus on PC axes that were significant predictor variables in final models and associated environmental variables with high loadings (>0.4; Table 3, summarized in Table 4).

**Table 4:** Predictor variables and *p*-values in final models of trip metrics relative to PC axes

Model variables	Dominant environmental variables in PCs	Farthest distance traveled	Trip duration	Proportion of Trips North of TZCF
Laysan Albatross- Incubating trips				
(Intercept)		NS	NS	NS
Black-footed Albatross-Incubating trips				
(Intercept)		2.00E-16	NS	NS
PC 2 _BFAL, incubating_	MEI (−), NPGO (+), Dist. TZCF (+)	4.60E-02		
R^2^		0.11		
Laysan Albatross-Brooding trips				
(Intercept)		2.00E-16	3.30E-16	2.00E-16
PC 1 _LAAL, brooding_	MEI (−), NPGO (+), Dist. TZCF (+), SST (+)	1.83E-02		4.62E-02
PC 2 _LAAL, brooding_	Dist. TZCF (+)	1.22E-02	4.60E-02	
R^2^		0.21	0.11	0.16
Black-footed Albatross-Brooding trips				
(Intercept)		2.00E-16	7.01E-11	NS
PC 1 _BFAL, brooding_	MEI (−), NPGO (+), Dist. TZCF (+), SST (−)	9.68E-08	4.20E-02	
R^2^		0.17	0.085	

NS indicates that final models were not significant (*p* > 0.05). Relationships with dominant environmental variables (loadings >0.3) are shown for each PC.

**Fig. 4:**
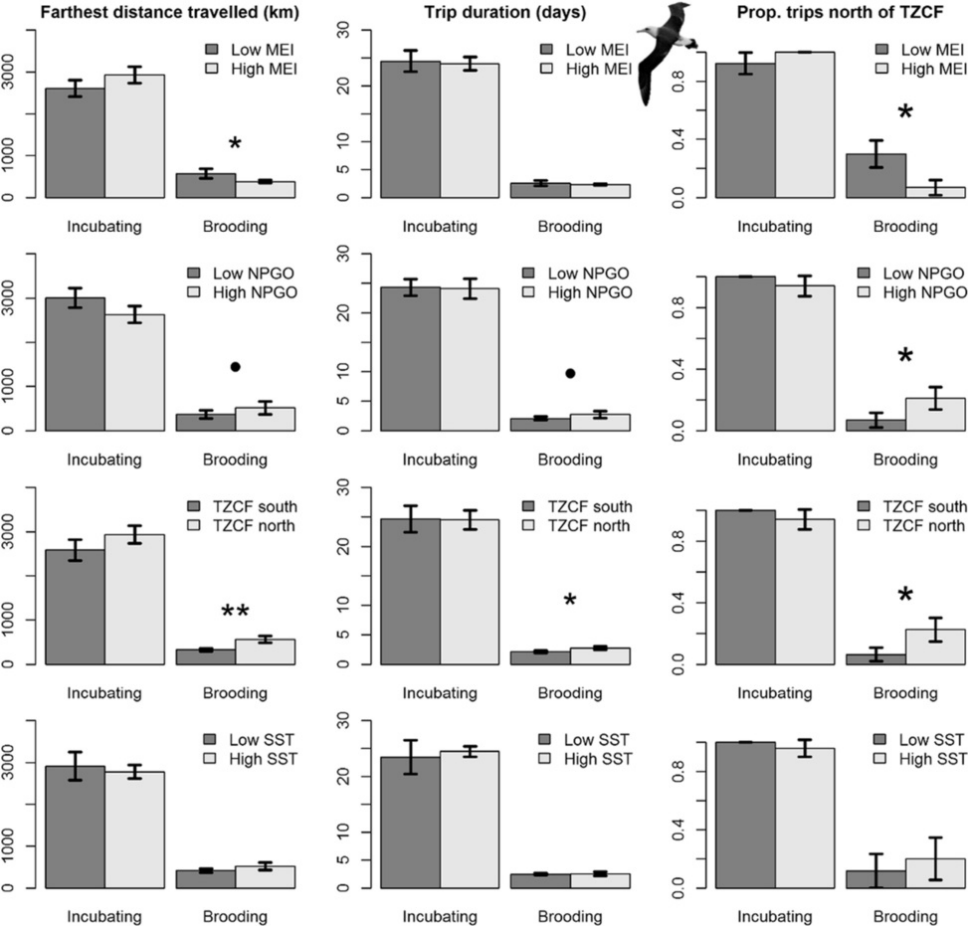
Laysan albatross trip metrics relative to environmental variables used in analyses. Low (high) values of environmental variables represent values lower (higher) than the mean. Comparisons were conducted using Wilcoxon signed rank tests; * indicates *p* values < 0.05, • indicates *p* values < 0.10

**Fig. 5:**
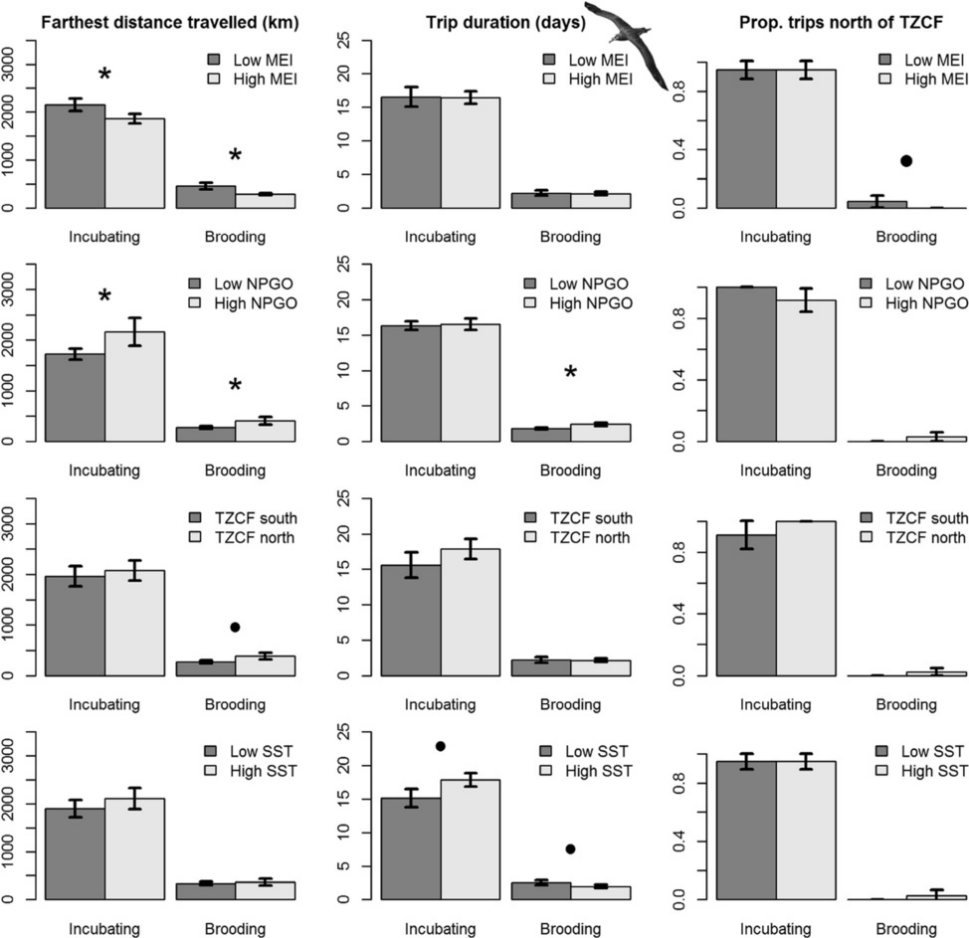
Black-footed albatross trip metrics relative to environmental variables used in analyses. Low (high) values of environmental variables represent values lower (higher) than the mean. Comparisons were conducted using Wilcoxon signed rank tests; * indicates *p* values < 0.05, • indicates *p* values < 0.10

Models of brooding Laysan albatross trips generally performed well, explaining 11–21 % of the variation in trip metrics, and highlighted the importance of large-scale climate variables. Brooding Laysan albatrosses traveled farther and traveled north of the TZCF more frequently during La Niña conditions, when NPGO was high and when the TZCF was farther north. When the TZCF was farther north, brooding Laysan albatrosses took longer trips (Table 4, Fig. 3). Similar trends were observed for Black-footed albatrosses, with models explaining 9–17 % of variability in trip metrics. Brooding Black-footed albatrosses traveled farther during La Niña conditions, when NPGO was high and when the TZCF was farther north, and took longer trips when NPGO was high. During the brooding period, Black-footed albatrosses rarely traveled north of the TZCF (1 of 56 birds tracked); consequently, models of trips north of the TZCF were not significant (Table 4). Oceanographic and climate variables did not have significant impacts on incubating Laysan albatrosses (Table 4, Fig. 4). Incubating Black-footed albatrosses traveled farther during La Niña conditions when NPGO was high (Table 4, Fig. 5).

### Oceanographic and climate effects on reproductive success

**Fig. 6:**
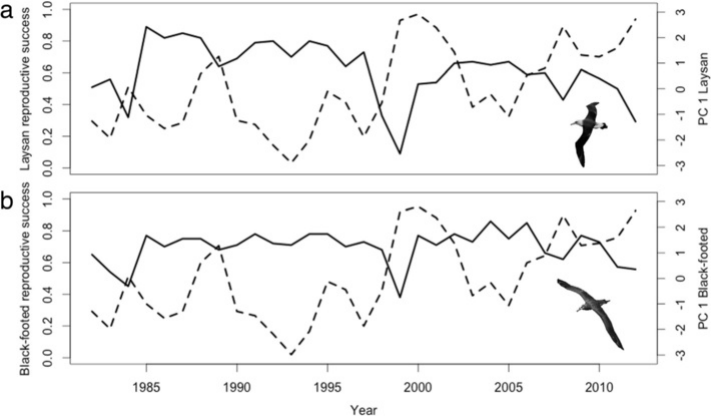
Reproductive success relative to PC1_annual_ scores for Laysan (**a**) and Black-footed albatrosses (**b**). Solid lines represent reproductive success, while dashed lines represent PC1_annual_ axes

At an annual scale, models of reproductive success performed relatively well for Laysan albatrosses, explaining 35 % of the variability in the data across the 31-year time series (Table 5). Laysan albatross reproductive success was negatively correlated with minimum distance to TZCF, SST and NPGO, and positively correlated with and MEI (Tables 3 and 5). While the GLM for Black-footed albatross reproductive success showed similar relationships, the model explained only 11 % of the variation. Four years (1984, 1999, 2008 and 2012) showed particularly low reproductive success, and three of these years (1999, 2008 and 2012) represented the three highest loadings along PC_ annual_ axis 1 for both species (Fig. 6). These three years represented La Niña conditions and/or conditions in which NPGO and SST were high and the TZCF was farther from Tern Island. The TZCF was typically farther away than the mean frontal location in years with low albatross reproductive success; however, this was not true in 1984.

**Table 5:** Predictor variables and *p*-values in final models of albatross reproductive success relative to PC axes

Model variables	Dominant environmental variables in PCs	Reproductive success
Laysan albatross		
Intercept		2.00E-16
PC1_LAAL, annual_	MEI (+), NPGO (−), Dist. TZCF (−), SST (−)	2.00E-16
R^2^		0.35
Black-footed albatross		
Intercept		2.00E-16
PC1_BFAL, annual_	MEI (+), NPGO (−), Dist. TZCF (−), SST (−)	9.90E-04
R^2^		0.11

**Fig. 7:**
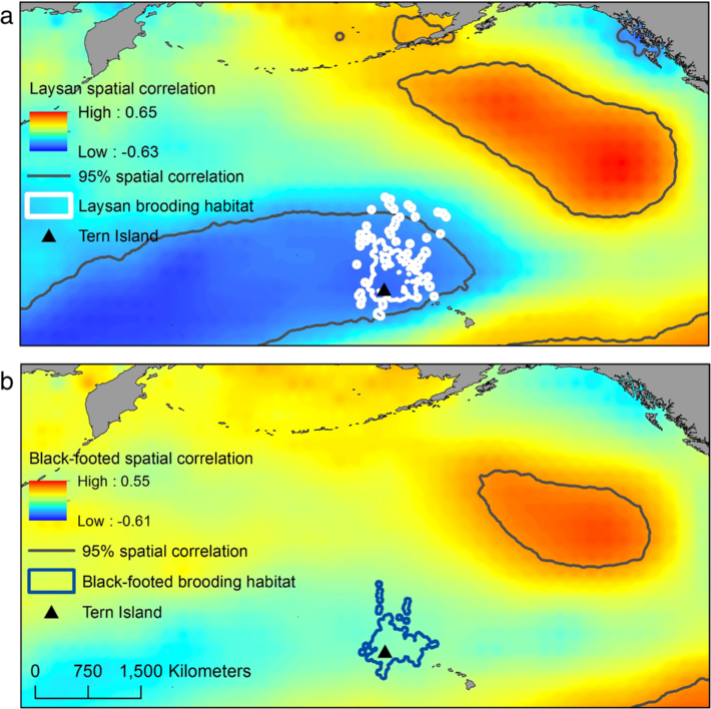
Spatial correlations between (**a**) Laysan and (**b**) Black-footed albatross reproductive success and SSTa. Correlation coefficients were calculated on an annual scale using November–February SSTa and albatross reproductive success. The solid black lines denote the threshold value for the 95 % confidence level. Brooding habitat is shown as a reference for both species

Figure 7 shows the spatial distribution of correlation coefficients between the time series of albatross reproductive success and each grid point of November–February average SSTa. Within Laysan albatross brooding habitat, there were strong negative correlations (>95 % confidence level) between Laysan albatross reproductive success and SSTa. Correlations with Black-footed albatross reproductive success within brooding habitat for this species were weaker (<95 % confidence level).

**Fig. 8:**
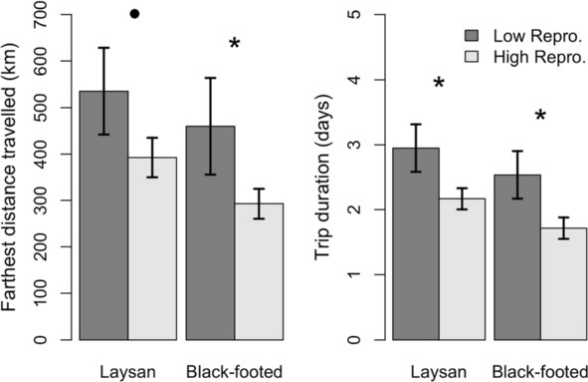
Relationships between brooding trip metrics and reproductive success for Laysan albatrosses and Black-footed albatrosses. Low (high) values of reprodcutive success represent values lower (higher) than the mean. Comparisons were conducted using Wilcoxon signed rank tests; * indicates *p* values < 0.05, • indicates *p* values < 0.10

At an annual scale, brooding trip metrics were correlated with reproductive success for both Laysan and Black-footed albatrosses. Farthest distance traveled and trip duration were negatively correlated with reproductive success during the brooding period (Fig. 8).

## Discussion

### Effects of environmental variability on albatross foraging trips

Our model results supported our hypothesis that changes in the foraging behavior of Laysan and Black-footed albatrosses breeding at Tern Island are dependent on the location of the TZCF and large-scale climatic variability. Lower MEI values (La Niña conditions) and higher NPGO values (expansion of North Pacific gyre) were correlated with increased distance to the TZCF, and were associated with longer trip distances for brooding albatrosses. Brooding albatrosses are often unable to reach the productive waters of the subarctic gyre north of the TZCF since they must return to the nest every few days to provision chicks. However, the location of the TZCF influences productivity in the region south of the front, where brooding albatrosses forage; when the front is farther south, primary productivity and presumably also associated prey distributions increase in waters south of the front [36]. Therefore, as their foraging habitat moves in association with the TZCF, brooding albatrosses forage farther from Tern Island when the TZCF is farther north, taking longer trips to reach their foraging habitat (Figs. 3 and 4).

SST is often used as a proxy for oceanographic conditions, with low SST representing improved foraging conditions for seabirds [e.g., 18, 60, 61]. La Niña conditions were generally associated with increased SST and a more northerly location of the TZCF (Fig. 3), reflecting poor foraging conditions for foraging albatrosses. Both species took particularly distant and long brooding trips during 2008, the strongest La Niña conditions during our tagging study. Our results suggest that albatrosses must travel farther to find food during La Niña conditions, particularly during the more spatially constrained brooding period.

### La Niña conditions linked to decreases in albatross reproductive success

Our results demonstrate how environmental variability influences the reproductive success of Laysan and Black-footed albatrosses breeding at Tern Island. Climatic and oceanographic factors that were associated with increases in trip length and range in brooding albatrosses (La Niña conditions, northward displacement of the TZCF, increased NPGO) were also associated with decreased reproductive success. Lower reproductive success was observed when the North Pacific subtropical gyre appeared to be in a period of expansion (positive NPGO) and, consequently, when the TZCF was farther away from Tern Island, representing a farther distance to travel to foraging grounds. Years with particularly low values of reproductive success in both study species were associated with extreme values in climatic and oceanographic variables, typically representing La Niña conditions. The TZCF was farther north (farther from Tern Island) in 1998, 2008 and 2012, which were years exhibiting dramatic declines in albatross reproductive success (Fig. 6). In contrast, both Laysan and Black-footed albatrosses showed a marked decline in reproductive success in 1984, a year which was not found to be anomalous in terms of environmental conditions. This year followed a very strong El Niño in 1982–1983 and represented a transition to La Niña conditions.

Spatial correlations between albatross reproductive success and annual SSTa highlight the importance of considering spatial patterns when linking marine predators with climate-driven environmental change. Maps of spatial correlations indicated that associations between albatross reproductive success and SSTa vary dramatically in the North Pacific, making it critical to link movement and foraging habitat with demographic trends, particularly for wide-ranging predators. Negative correlations between SSTa and reproductive success were particularly strong within brooding habitat for Laysan’s albatross (Fig. 7), providing further support for the importance of the brooding stage in understanding climate impacts on this species.

While El Niño events have been linked with increases in SST and decreased foraging success and declining population trends of marine predators in the California Current [e.g., 57–59], La Niña events were associated with increased SST in the central North Pacific and decreases in albatross reproductive success at Tern Island. Monk seals in this area also rely on the TZCF for foraging and have shown similar relationships, with increases in body condition and survival during El Niño events [38].

### Foraging behavior linked to decreases in albatross reproductive success

Higher trip distance and duration during brooding trips were associated with decreased reproductive success (Fig. 8), indicating the importance of maximizing food delivery to the chick during this period. Brooding trip distance and duration showed positive relationships with poor foraging conditions (La Niña conditions, expansion of the North Pacific gyre, and/or increased distance to the TZCF), suggesting increased energy expenditure by brooding birds during poor conditions negatively impacts reproductive success. Albatross are long-lived birds that maintain their own body condition over the survival of their chicks [e.g., 62] and our findings suggest that high energetic costs of brooding trips during poor foraging conditions may cause adults to abandon their nests.

### Laysan albatrosses show strong responses to poor foraging conditions

**Fig. 9:**
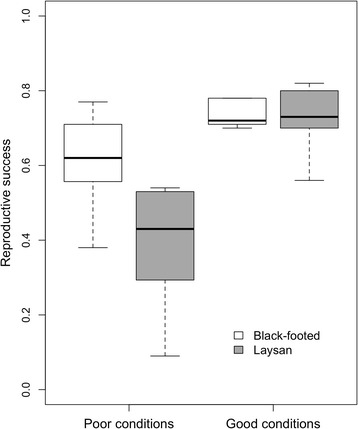
Black-footed albatross and Laysan albatross reproductive success during poor and good conditions, respectively. Good and bad conditions are represented by the five years with the lowest and highest PC1_annual_ scores, respectively, for each species

Reproductive success was higher in Black-footed albatrosses than in Laysan albatrosses during poor conditions despite increases in the distance and duration of brooding trips; during good conditions, the reproductive success of both species was similar (Fig. 9). This suggests that Black-footed albatrosses are better suited to dealing with variable environmental conditions, while Laysan albatrosses perform better (demonstrating a higher reproductive success relative to the 31-year mean for this species) when environmental conditions are favorable. When unconstrained by brooding requirements, the foraging habitat of Laysan albatrosses is farther north than that of Black-footed albatrosses [29, 33] and Laysan albatrosses frequently travel north of the TZCF during incubating trips (Figs. 1 and 4). Our results suggest that this more distant foraging habitat is generally unreachable to Laysan albatrosses during the more constraining brooding period. For Laysan albatrosses, temporal constraints of the brooding period combined with an increased distance to high latitude foraging appear to contribute to the mean overall lower reproductive success observed for this species grounds in comparison to Black-footed albatrosses (0.62 +/−0.18 chicks fledged per eggs laid for Laysan albatrosses, 0.70 +/−0.11 chicks fledged per eggs laid for Black-footed albatrosses from 1981/1982–2011/2012).

### Effects of wind variability on the accessibility of foraging habitat

Recent studies have indicated the importance of wind speed and direction to albatross populations [19, 64]. Increases in reproductive success and poleward shifts in foraging range in Southern Ocean albatrosses have been linked with stronger winds that have moved poleward [19]. Though overall conditions for foraging albatrosses appear to be suboptimal during La Niña events in the central North Pacific (higher SST, increased distance to TZCF), stronger trade winds during La Niña events [63] may provide energetic benefits. Black-footed albatrosses, likely more limited in their foraging ranges due to higher wing-loading [64], may take advantage of increased trade winds during La Niña events to travel farther with lower energy requirements. Future work will focus on quantifying and comparing the effects of wind direction and intensity on the foraging movements of brooding Laysan and Black-footed albatrosses in order to evaluate this hypothesis in detail.

## Conclusions

Though many studies have linked seabird foraging behavior or reproductive success with environmental factors such as SST [e.g., 14, 65–68], few studies have quantified the behavioral mechanisms that underlie population responses to environmental variability. Our results demonstrate how oceanographic and climatic factors influence albatross foraging behavior, and how this translates to reproductive success. These links appear to be mediated through constraints on movement and time at sea during the brooding phase, when increased energetic demands associated with frequent feedings of chicks imposes limits on the duration of foraging trips of the parents. The location of the TZCF, NPGO state, and ENSO conditions in the central Pacific were found to be important drivers of these responses. Increases in foraging range and duration in response to environmental conditions were associated with lowered reproductive success (Fig. 8).

Our findings highlight the importance of habitat shifts in response to climate-induced environmental change and the potential for these shifts to influence populations of marine predators. Climate change predictions for the North Pacific suggest that changes to albatross habitat may be amplified in future years. The TZCF has shifted farther north over the past 30 years as the subtropical gyre has expanded [Thorne, unpublished data], which has likely reduced accessibility to preferred feeding grounds for breeding albatrosses. For example, we estimated that the TZCF was within the mean flight range of a brooding Laysan albatross during 17 % of days during the brooding period from 1982–1991 compared to only 3 % of days from 2002–2012. Climate models suggest that this northward trend will continue in the future and that SST in the expanding subtropical biome will increase dramatically by the end of the century [39]. Our results suggest that these patterns will cause decreases in the reproductive output of albatrosses and reduced colony performance in the Northwest Hawaiian Islands, although these species will face cumulative effects from climate impacts, either muting or accelerating this decline. This study demonstrates the importance of elucidating links between oceanography, behavior and population change to understand likely ecosystem response to climate variability and change.

## References

[1] Pauly D, Christensen V, Dalsgaard J, Froese R, Torres F (1998). Fishing down marine food webs. Science.

[2] Stirling I, Lunn NJ, Iacozza J (1999). Long-term trends in the population ecology of polar bears in western Hudson Bay in relation to climatic change. Arctic.

[3] Baum JK, Myers RA, Kehler DG, Worm B, Harley SJ, Doherty PA (2003). Collapse and conservation of shark populations in the Northwest Atlantic. Science.

[4] Lewison RL, Crowder LB, Read AJ, Freeman SA (2004). Understanding impacts of fisheries bycatch on marine megafauna. Trends Ecol Evol.

[5] Williams TM, Estes JA, Doak DF, Springer AM (2004). Killer appetites: assessing the role of predators in ecological communities. Ecology.

[6] Myers RA, Baum JK, Shepherd TD, Powers SP, Peterson CH (2007). Cascading effects of the loss of apex predatory sharks from a coastal ocean. Science.

[7] Heithaus MR, Frid A, Wirsing AJ, Worm B (2008). Predicting ecological consequences of marine top predator declines. Trends Ecol Evol.

[8] Estes JA, Terborgh J, Brashares JS, Power ME, Berger J, Bond WJ (2011). Trophic downgrading of planet Earth. Science.

[9] Roman J, Estes JA, Morissette L, Smith C, Costa D, McCarthy J (2014). Whales as marine ecosystem engineers. Front Ecol Environ.

[10] Hazen EL, Jorgensen S, Rykaczewski RR, Bograd SJ, Foley DG, Jonsen ID (2013). Predicted habitat shifts of Pacific top predators in a changing climate. Nat Clim Chang.

[11] IPCC. 2007: Synthesis Report (eds Core Writing Team, Pachauri, R. K. & Reisinger, A.) (IPCC, 2007); available at www.ipcc.ch/pdf/assessment-report/ar4/syr/ar4_syr.pdf. 2007.

[12] Hoegh-Guldberg O, Bruno JF (2010). The impact of climate change on the world’s marine ecosystems. Science.

[13] Péron C, Weimerskirch H, Bost C-A (2012). Projected poleward shift of king penguins’ (Aptenodytes patagonicus) foraging range at the Crozet Islands, southern Indian Ocean. Proc Biol Sci.

[14] Croxall JP, Trathan P, Murphy E (2002). Environmental change and Antarctic seabird populations. Science.

[15] Regehr EV, Hunter CM, Caswell H, Amstrup SC, Stirling I (2010). Survival and breeding of polar bears in the southern Beaufort Sea in relation to sea ice. J Anim Ecol.

[16] Moore SE, Huntington HP (2008). Arctic marine mammals and climate change: impacts and resilience. Ecol Appl.

[17] Forcada J, Trathan PN (2009). Penguin responses to climate change in the Southern Ocean. Glob Chang Biol.

[18] Inchausti P, Guinet C, Koudil M, Durbec JP, Barbraud C, Weimerskirch H (2003). Inter‐annual variability in the breeding performance of seabirds in relation to oceanographic anomalies that affect the Crozet and the Kerguelen sectors of the Southern Ocean. J Avian Biol.

[19] Weimerskirch H, Louzao M, De Grissac S, Delord K (2012). Changes in wind pattern alter albatross distribution and life-history traits. Science.

[20] Montevecchi W, Myers A (1996). Dietary changes of seabirds indicate shifts in pelagic food webs. Sarsia.

[21] Montevecchi W, Myers R (1997). Centurial and decadal oceanographic influences on changes in northern gannet populations and diets in the north-west Atlantic: implications for climate change. ICES J Mar Sci.

[22] Cairns D (1988). Seabirds as indicators of marine food supplies. Biological Oceanography.

[23] Piatt J, Sydeman W, Sydeman W, Piatt J, Browman H (2007). Seabirds as indicators of marine ecosystems. Mar Ecol Prog Ser.

[24] Costa D, Prince P (1987). Foraging energetics of Grey‐headed Albatrosses Diotnedea chrysostoma at Bird Island, South Georgia. Ibis.

[25] Weimerskirch H, Guionnet T, Martin J, Shaffer SA, Costa D (2000). Fast and fuel efficient? Optimal use of wind by flying albatrosses. Proc Roy Soc Lond B Biol Sci.

[26] Shaffer SA, Costa DP, Weimerskirch H (2001). Behavioural factors affecting foraging effort of breeding wandering albatrosses. J Anim Ecol.

[27] Weimerskirch H, Ancel A, Caloin M, Zahariev A, Spagiari J, Kersten M (2003). Foraging efficiency and adjustment of energy expenditure in a pelagic seabird provisioning its chick. J Anim Ecol.

[28] Weimerskirch H, Salamolard M, Sarrazin F, Jouventin P (1993). Foraging strategy of wandering albatrosses through the breeding season: a study using satellite telemetry. The Auk.

[29] Hyrenbach KD, Fernández P, Anderson DJ (2002). Oceanographic habitats of two sympatric North Pacific albatrosses during the breeding season. Mar Ecol Prog Ser.

[30] Shaffer SA, Costa DP, Weimerskirch H (2003). Foraging effort in relation to the constraints of reproduction in free‐ranging albatrosses. Functional Ecology.

[31] Shaffer SA (2004). Annual energy budget and food requirements of breeding wandering albatrosses (Diomedea exulans). Polar Biology.

[32] Bevan R, Butler P, Woakes A, Prince P (1995). The energy expenditure of free-ranging black-browed albatrosses. Philos Trans R Soc Lond B Biol Sci.

[33] Kappes MA, Shaffer SA, Tremblay Y, Foley DG, Palacios DM, Robinson PW (2010). Hawaiian albatrosses track interannual variability of marine habitats in the North Pacific. Prog Oceanogr.

[34] Wakefield ED, Phillips RA, Matthiopoulos J (2014). Habitat-mediated population limitation in a colonial central-place forager: the sky is not the limit for the black-browed albatross. Proc Biol Sci.

[35] Polovina JJ, Howell E, Kobayashi DR, Seki MP (2001). The transition zone chlorophyll front, a dynamic global feature defining migration and forage habitat for marine resources. Prog Oceanogr.

[36] Baker JD, Polovina JJ, Howell EA (2007). Effect of variable oceanic productivity on the survival of an upper trophic predator, the Hawaiian monk seal Monachus schauinslandi. Mar Ecol Prog Ser.

[37] Bograd SJ, Foley DG, Schwing FB, Wilson C, Laurs RM, Polovina JJ (2004). On the seasonal and interannual migrations of the transition zone chlorophyll front. Geophys Res Lett.

[38] Antonelis GA, Baker JD, Polovina JJ (2003). Improved body condition of weaned Hawaiian monk seal pups associated with El Niño events: Potential benefits to an endangered species. Mar Mamm Sci.

[39] Polovina JJ, Dunne JP, Woodworth PA, Howell EA (2011). Projected expansion of the subtropical biome and contraction of the temperate and equatorial upwelling biomes in the North Pacific under global warming. ICES J Mar Sci.

[40] Fernandez P, Anderson DJ, Sievert PR, Huyvaert KP (2001). Foraging destinations of three low-latitude albatross (Phoebastria) species. J Zool.

[41] Whittow GC, Pooley A, Gill F (1983). The black-footed albatross (Diomedea nigripes). The birds of North America.

[42] Whittow GC, Pooley A, Gill F (1993). The Laysan albatross (Diomedea immutabilis). The birds of North America.

[43] Arata JA, Sievert PR, Naughton MB. Status assessment of Laysan and black-footed albatrosses, North Pacific Ocean, 1923–2005. US Geological Survey Scientific Investigations Report. 2009;5131. 80 p.

[44] Fisher HI (1971). The Laysan albatross: its incubation, hatching, and associated behaviors. Living Bird.

[45] Fisher HI (1975). Mortality and survival in the Laysan albatross, *Diomedea immutabilis*. Pac Sci.

[46] Phillips RA, Xavier JC, Croxall JP, Burger A (2003). Effects of satellite transmitters on albatrosses and petrels. The Auk.

[47] Flemming JM, Jonsen ID, Myers RA, Field CA (2010). Hierarchical state-space estimation of leatherback turtle navigation ability. PLoS One.

[48] R Core Team (2013). R: A language and environment for statistical computing.

[49] Di Lorenzo E, Cobb K, Furtado J, Schneider N, Anderson B, Bracco A (2010). Central Pacific El Niño and decadal climate change in the North Pacific Ocean. Nat Geosci.

[50] Di Lorenzo E, Schneider N, Cobb K, Franks P, Chhak K, Miller A (2008). North Pacific Gyre Oscillation links ocean climate and ecosystem change. Geophys Res Lett.

[51] Wolter K, Timlin MS (1998). Measuring the strength of ENSO events: how does 1997/98 rank?. Weather.

[52] Shillinger GL, Bailey H, Bograd SJ, Hazen EL, Hamann M, Gaspar P (2012). Tagging through the stages: technical and ecological challenges in observing life histories through biologging. Mar Ecol Prog Ser.

[53] McCullagh P, Nelder JA (1989). Generalized linear models.

[54] Engler R, Guisan A, Rechsteiner L (2004). An improved approach for predicting the distribution of rare and endangered species from occurrence and pseudo‐absence data. J Appl Ecol.

[55] Akaike H (1973). Maximum likelihood identification of Gaussian autoregressive moving average models. Biometrika.

[56] Burnham K, Anderson D (1998). Model selection and inferences.

[57] Chavez F, Pennington J, Castro C, Ryan J, Michisaki R, Schlining B (2002). Biological and chemical consequences of the 1997–1998 El Niño in central California waters. Prog Oceanogr.

[58] Crocker DE, Costa DP, Le Boeuf BJ, Webb PM, Houser DS. Impact of El Niño on the foraging behavior of female northern elephant seals. Mar Ecol Prog Ser. 2006;309.

[59] Lee DE, Nur N, Sydeman WJ (2007). Climate and demography of the planktivorous Cassin’s auklet Ptychoramphus aleuticus off northern California: implications for population change. J Anim Ecol.

[60] Guinet C, Chastel O, Koudil M, Durbec JP, Jouventin P (1998). Effects of warm sea–surface temperature anomalies on the blue petrel at the Kerguelen Islands. Proc Roy Soc Lond B Biol Sci.

[61] Peck DR, Smithers BV, Krockenberger AK, Congdon BC (2004). Sea surface temperature constrains wedge-tailed shearwater foraging success within breeding seasons. Mar Ecol Prog Ser.

[62] Weimerskirch H, Zimmermann L, Prince PA (2001). Influence of environmental variability on breeding effort in a long-lived seabird, the yellow-nosed albatross. Behav Ecol.

[63] Schwing F, Murphree T, Dewitt L, Green PM (2002). The evolution of oceanic and atmospheric anomalies in the northeast Pacific during the El Niño and La Niña events of 1995–2001. Prog Oceanogr.

[64] Suryan RM, Anderson DJ, Shaffer SA, Roby DD, Tremblay Y, Costa DP (2008). Wind, waves, and wing loading: morphological specialization may limit range expansion of endangered albatrosses. PLoS One.

[65] Ballance LT, Pitman RL, Fiedler PC (2006). Oceanographic influences on seabirds and cetaceans of the eastern tropical Pacific: a review. Prog Oceanogr.

[66] Becker BH, Peery M, Beissinger SR (2007). Ocean climate and prey availability affect the trophic level and reproductive success of the marbled murrelet, an endangered seabird. Mar Ecol Prog Ser.

[67] Frederiksen M, Edwards M, Mavor RA, Wanless S (2007). Regional and annual variation in black-legged kittiwake breeding productivity is related to sea surface temperature. Mar Ecol Prog Ser.

[68] Wolf SG, Sydeman WJ, Hipfner JM, Abraham CL, Tershy BR, Croll DA (2009). Range-wide reproductive consequences of ocean climate variability for the seabird Cassin’s Auklet. Ecology.

